# Binding Modes of Three Inhibitors 8CA, F8A and I4A to A-FABP Studied Based on Molecular Dynamics Simulation

**DOI:** 10.1371/journal.pone.0099862

**Published:** 2014-06-11

**Authors:** Jianzhong Chen, Jinan Wang, Weiliang Zhu

**Affiliations:** 1 School of Science, Shandong Jiaotong University, Jinan, China; 2 Discovery and Design Center, CAS Key Laboratory of Receptor Research, Shanghai Institute of Materia Medica, Chinese Academy of Sciences, Shanghai, China; University of Akron, United States of America

## Abstract

Adipocyte fatty-acid binding protein (A-FABP) is an important target of drug designs treating some diseases related to lipid-mediated biology. Molecular dynamics (MD) simulations coupled with solvated interaction energy method (SIE) were carried out to study the binding modes of three inhibitors 8CA, F8A and I4A to A-FABP. The rank of our predicted binding affinities is in accordance with experimental data. The results show that the substitution in the position 5 of *N*-benzyl and the seven-membered ring of *N*-benzyl-indole carboxylic acids strengthen the I4A binding, while the substitution in the position 2 of *N*-benzyl weakens the F8A binding. Computational alanine scanning and dynamics analyses were performed and the results suggest that the polar interactions of the positively charged residue R126 with the three inhibitors provide a significant contribution to inhibitor bindings. This polar interaction induces the disappearance of the correlated motion of the C terminus of A-FABP relative to the N terminus and favors the stability of the binding complex. This study is helpful for the rational design of potent inhibitors within the fields of metabolic disease, inflammation and atherosclerosis.

## Introduction

Fatty acid binding proteins are small cytoplasmic proteins that are expressed in a tissue-specific manner [1]. It can bind to fatty acids such as oleic and retinoic acid, and transport these fatty acids from cellular surface to multiform sites of metabolism or storage [2]. FABPs involve lipid-mediated biology such as signaling pathways trafficking and membrane synthesis [3], [4].

Adipocyte FABP is one of the nine known FABP isoforms, and highly expressed in adipose tissue and macrophages [5]. The previous published works show that A-FABP can perform an important function in certain specific aspects of the metabolic syndrome and cardiovascular disease [6]–[8]. Some studies on A-FABP function of mouse model suggested that functional disruption and deletion of A-FABP reduce risk of atherosclerosis in apolipoprotein E-deficient mice [1], [6], [9], and also inhibit development of diet-induced insuline resistence [3], [6], [10]. Reductions of A-FABP in adipose issue of human induced a lower risk of hypertriglyceridemia, type 2 diabetes and coronary heart disease [11]–[13]. Thus, A-FABP was considered as an important target of drug designs treating some diseases related to lipid-mediated biology.

Pharmacological intervention of A-FABP functions could play an therapeutic role in disorders such as type 2 diabetes and atherosclerosis [7], [14]. An selective A-FABP inhibitor BMS309403 produced protection of atherosclerosis and diabetics in mouse model [11]. Scarce literature on small molecule inhibitors for this family of protein showed potential of pharmacological intervention [14]–[16]. Design of small molecule inhibitors of A-FABP aroused significant interest in drug treatment in the fields of metabolic disease, inflammation of and atherosclerosis [17], [18].

Barf et al. clarified the structure-activity relationship of inhibitor/A-FABP complex by using carbazole- and indole-based inhibitors of A-FABP, resulting in the discovery of submicromolar inhibitors [16]. They also performed optimization on new benzoic acid scaffolds to identify several ligands with nanomolar potency [17]. These studies show possibility of developing potent inhibitors of A-FABP, also remove concerns on the possibility to develop isoform selective compounds, the lipophilic and charged nature of the endogenous ligands and how this translates to the drugability of the binding pocket. Thus, it is significant to clarify binding mechanism of small molecular inhibitors to A-FABP and understand internal dynamics of A-FABP induced by inhibitor bindings for development of potent A-FABP inhibitors.

**Figure 1:**
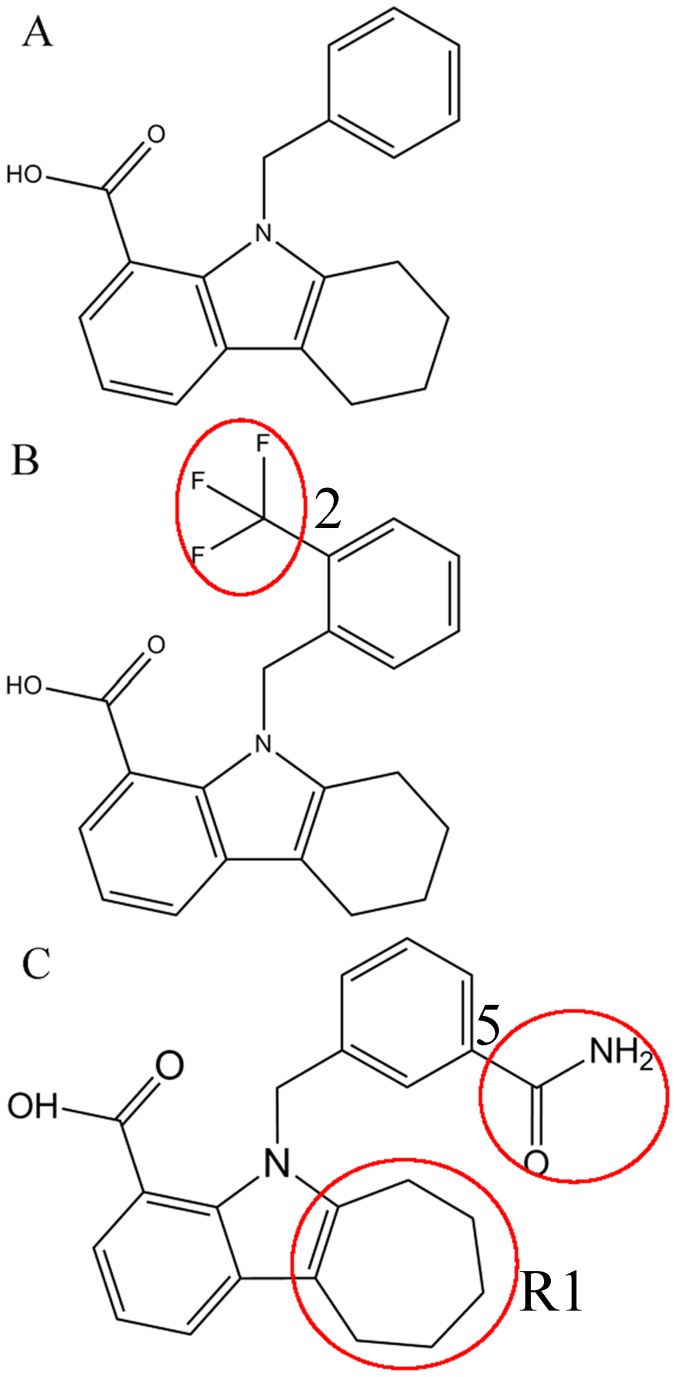
Molecular structures of the three inhibitors 8CA (A), F8A(B) and I4A(C). The structural difference is labeled by red circle.

Molecular dynamics (MD) simulations and calculations of binding free energies have been a powerful tool of insight into interactions of inhibitors with proteins [19]–[30]. Cross-correlation analysis based on MD trajectory is also an efficient means probing internal motions in proteins [31]–[33]. In this work, three small molecular inhibitors 8CA, F8A and I4A were selected to study their binding mechanism to A-FABP at an atomic level [17]. The three inhibitors share a common scaffold with *N*-benzyl-indole carboxylic acids (Fig. 1). The inhibitors F8A and I4A are the derivatives of the substitutions in the position 2 and 5 of *N*-benzyl, respectively. Moreover, the ring R1 of the scaffold is replaced by a seven-membered ring in I4A. The understanding of difference in binding modes induced by these three structurally different inhibitors is significant for the rational design of potent inhibitors. Thus, in this study, various simulation techniques, including MD simulations, solvated interaction energy method, computational alanine scanning and cross-correlation analysis will be integrated to probe the binding modes of the three inhibitors to A-FABP. We also expected that this study can theoretically contribute a significant guidance to the design of potent drugs targeting A-FABP.

## Methods

### Starting Structures

The initial coordinates of 8CA, F8A and I4A/A-FABP complexes were obtained from the protein data bank and their PDB entry are 3FR2, 3FR4 and 3FR5, respectively [17]. All crystal water molecules were retained in the starting model. FF03 force field was used to produce the parameters of protein and water molecules [34]. The general amber force field was assigned to the three inhibitors [35]. The am1-bcc method implemented in Amber12 was applied to assign the partial atomic charges to the three inhibitors [36], [37]. The side-chain protonation states were assigned at PH = 7.0 by using PROPKA program [38], [39]. Each system was solvated in a truncated octahedron box of TIP3P water molecules with a 12.0 Å buffer along each dimension [40]. An appropriate number of sodion counterions were added to produce a neutral charge on the system.

### Molecular Dynamics Simulations

For each system, energy minimizations and MD simulations were carried out using the sander module in Amber12 program [37]. To remove bad contacts between the complex and solvent molecules, each system was subject to energy minimizations in two stages. Firstly, the water molecules and counterions were minimized by freezing the complex using a harmonic constraint of a strength of 100 kcal·mol^−1^·Å^−2^. Secondly, all atoms were energy-minimized without restriction. And each stage was performed using the steepest descent minimization of 2000 steps followed by a conjugate gradient minimization of 4000 steps. Then, the system was heated from 0 to 300 K in 500 ps and equilibrated at 300 K for another 500 ps. Finally, a 60 ns simulation without restriction was conducted at constant pressure and 300 K, and the coordinates were saved every 2 ps. During MD simulations, the SHAKE method was applied to constraint the covalent bonds involving hydrogen atom [41]. The Particle Mesh Ewald (PME) method was adopted to treat the long-range electrostatic interactions [42], [43]. The cutoff distances for the long-range electrostatic and van der Waals interactions were set to 10.0 Å.

### Calculations of Binding Free Energies

Currently, free energy perturbation (FEP), thermodynamic integration (TI), molecular mechanics Poisson-Boltzmann surface area (MM-PSA) and solvated interaction energy methods etc. are usually used to calculate binding free energies. Although FEP and TI methods should give more accurate results, they are extremely time-consuming and require sufficient statistical samplings [44]–[46]. In MM-PBSA method, normal-mode analysis used to calculate the entropy change also endures heavy computational cost [47]. Thus, in this work, SIE method that can provide fast and rational calculation was adopted to compute the binding free energies [48]. This method has been successfully applied to predict the binding modes for dopamine D2 receptor to inhibitors [49], and also performed on the study of protein-protein interactions for the MP1-p14 scaffolding complex [50]. In this work, 200 snapshots extracted from the last 20-ns MD trajectory of the inhibitor/A-FABP complex at an interval of 100 ps were used for the binding free energy analyses. The SIE function [48] to calculate inhibitor-protein free energy is expressed as following
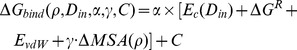
(1)where 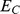 and 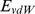 represent the intermolecular Coulomb and van der Waals interaction energies in the bound state, respectively. These two terms were calculated using the Amber molecular mechanics force field ff03 [34]. 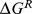 is the change in the reaction field energy induced by inhibitor binding and was calculated by solving the Poisson equation with the boundary element method BRI BEM [51], [52] and a variable-radius solvent probe [53]. The term 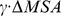 corresponds to the change in the molecular surface area upon binding. The parameters 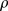, 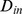, 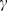 and 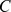 are Amber van der Waals radii linear scaling coefficient, the solute interior dielectric constant, the molecular surface area coefficient and a constant, respectively. The parameter 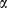 corresponds to the global proportionality coefficient related to the loss of conformational entropy upon binding [54]. The optimized values of these parameters are 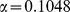, 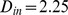, 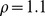, 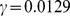 kcal/(mol·Å^2^) and 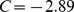 kcal·mol^−1^, respectively [48], [50]. The SIE calculations were performed with the program Sietraj [50].

### Computational Alanine Scanning

Computational alanine scanning method can be used to estimate the interactions of the side chain of residues in proteins with inhibitors. In this work, to examine the effect of electrostatic interactions of the residues Arg126 with inhibitors on bindings, alamine mutation was performed on Arg126. The alanine mutant structure was obtained by altering the coordinates of the wild-type residues, which involves cutting atoms and truncating the mutated residue at C_γ_ by replacing with a hydrogen atom [55]. All parameters in the topology file of the mutated residue were accordingly replaced by the alanine residue parameters. 60-ns MD simulations were carried out for three R126A-inhibitor complexes. 200 snapshots taken from the last 20 ns of MD trajectory with a time interval of 100 ps were applied to calculate the binding free energy.

### Cross-correlation Analysis

In folded proteins, the motions of many residues tend to be correlated. To investigate the effect of inhibitor binding on correlated motions of residues in A-FABP, the cross-correlation matrix 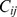, which reflects the fluctuation in the coordinates of C_α_ atoms relative to their average positions from the last 20 ns of the MD simulations, was calculated by the following equation [31], [56], [57]:
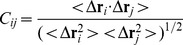
(2)in which 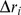 is the displacement from the mean position of the *i*th atom and the symbol<>represents time average over the MD trajectory. The values of 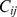 fluctuate from −1 to 1. Positive 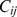 values indicate a correlated motion between the *i*th residue and the *j*th residue, while negative 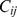 values represent an anti-correlated motion.

## Results and Discussion

### Root Mean Square Deviation of the C_α_ Atoms

**Figure 2:**
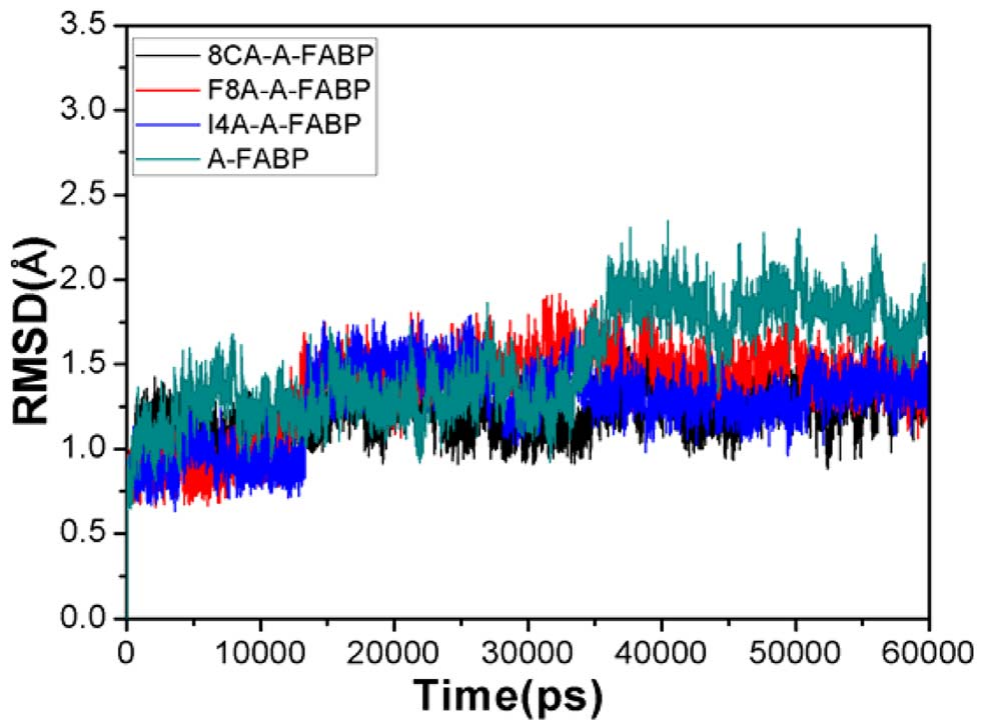
The root-mean-square deviation (RMSD) of the backbone atoms relative to their crystal structure as a function of time for 8CA (black), F8A(red), I4A(blue) and unbound A-FABP (dark cyan).

Stability of the complex structure can be reflected by its root mean square deviation (RMSD) of the C_α_ atoms from the initial structure. The RMSD values of four simulated systems along the entire MD trajectory are shown in Fig. 2. One can see that the time of three binding complexes reaching the equilibrium is shorter than unbound A-FABP, and the averaged RMSD values of three bound states of A-FABP to 8CA, F8A and I4A are 1.27, 1.45 and 1.31 Å, respectively, and also lower than unbound A-FABP (1.82 Å). This result implies that the three inhibitor bindings restrain the motions of some regions in A-FABP and favor the stability of the complex structure.

### Internal Dynamics

**Figure 3:**
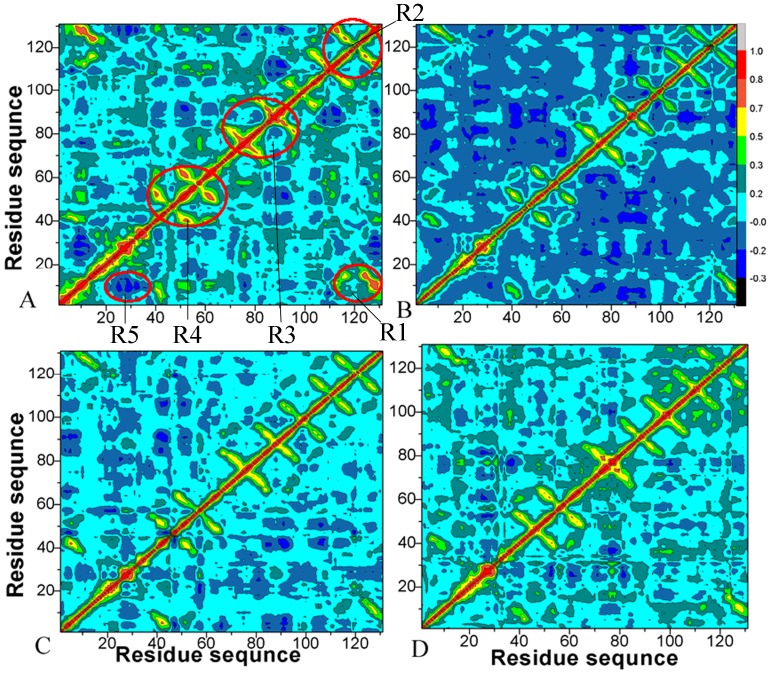
Cross-correlation matrices of the fluctuations of the coordinates for C_α_ atoms around their mean positions during the equilibrium phase of the simulations. The extent of correlated motions and anticorrelated motions are color-coded for unbound A-FABP(A), 8CA(B), F8A(C) and I4A(D).

To examine the effect of inhibitor bindings on correlated motions of residues in A-FABP, cross-correlation matrices of the fluctuation were calculated and plotted in Fig. 3. The extent of correlation in the movements between specific residues is shown in a color-coded manner.

The cross-correlation matrices (Fig. 3) show that global dynamics of the unbound form of A-FABP and the bound form are very similar to each other. This result suggests that inhibitor bindings do not highly change the original structure of A-FABP. However, major differences are still observed in some regions such as the regions R1, R2, R3, R4 and R5. These differences may reflect how inhibitor bindings induce the changes of protein’s local dynamics and conformational subspace. As seen in Fig. 3A, the strong correlated movements (red) occur in the diagonal regions R2, R3, R4 and R5 of unbound A-FABP, and the off-diagonal region R1 located in the C terminus of A-FABP also produces strong correlated motion relative to the residues 1–16 in the N terminus. These correlated motions disappear after inhibitor bindings (Fig. 3B–C). Additionally, inhibitor bindings also lead to the disappearance of anticorrelated motions between the region R5 and the residues 9–15. These results show that the regions R1–R5 may bear greater local conformational changes, also imply these regions may be the potential targets with which inhibitors interact. Structural information from PDB verifies that the regions R2–R5 are located nearby the loop, while the region R1 is located in the β strands (the residues 122–131) of the C terminus of A-FABP [17]. This result provides a hint that specific residues in the region R1 may produce strong interaction with inhibitors.

**Figure 4:**
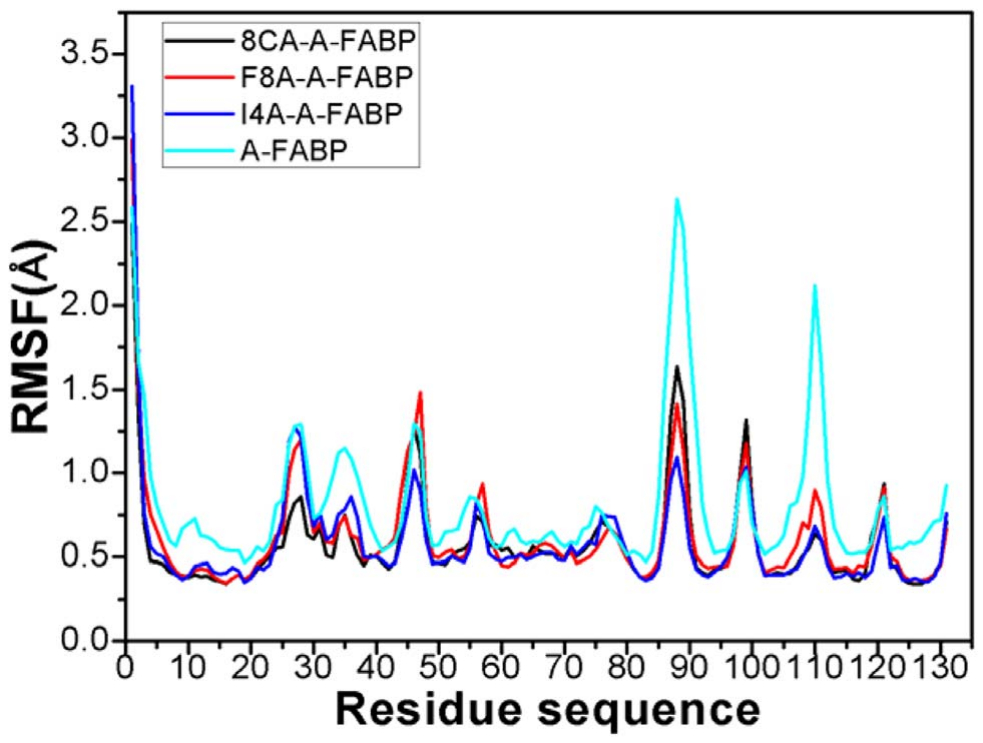
Plot of the RMSF of C_α_ atoms in A-FABP through the equilibrium phase of MD simulation.

To further understand the effect of inhibitor bindings on internal dynamics, the root mean square fluctuation (RMSF) of the C_α_ atoms was computed and displayed in Fig. 4. As seen from Fig. 4, inhibitor bindings produce obvious decrease of RMSF values in some key regions of A-FABP. These regions involve the residues 8–21, 32–36, 51–58, 86–94, 105–115 and 120–123, especially the regions 86–94, 105–115 and 120–123 are more obvious. In addition, inhibitor bindings also induce small reduction of RMSF values in a wide region of the residues 60–78. This result basically agrees with the above cross-correlation analysis, and also implies that some key residues in these regions may produce strong interactions with the three inhibitors.

### The Structure-affinity Relationship

The SIE method was applied to calculate binding free energies of the three inhibitors to A-FABP and the results were listed in Table 1. One can see that the binding free energies of the inhibitors 8CA, F8A and I4A to A-FABP are −8.63, −8.42 and −9.58 kcal·mol^−1^, respectively. It is encouraging that the rank of our predicted binding free energies is in agreement with the experimentally determined rank. Furthermore, most of the binding free energies even quantitatively agree with the experimental value. Exception is that the binding affinity of I4A is obviously higher than the experimental value.

**Table 1:** Binding free energies of wild-type and mutant A-FABP to inhibitors calculated by the SIE method^a^.

Energy^b^	8CA-wild	8CA-mutant	F8A-wild	F8A-mutant	I4A-wild	I4A-mutant
ΔE_vdw_	−33.35±0.19	−32.59±0.16	−33.29±0.12	−32.74±0.21	−41.61±0.22	−40.33±0.18
ΔE_c_	−57.40±0.24	−20.84±0.21	−52.04±0.24	−21.34±0.43	−59.51±0.23	−28.08±0.28
γ·ΔMSA	−7.55±0.22	−7.29±0.15	−7.89±0.02	−7.74±0.05	−8.79±0.02	−8.05±0.04
ΔG^R^	43.81±0.03	24.93±0.02	40.84±0.22	22.45±0.22	45.88±0.17	27.44±0.18
ΔG_bind_	−8.63±0.04	−6.59±0.02	−8.42±0.02	−6.97±0.04	−9.58±0.03	−7.90±0.03
ΔG^exp^	−8.52		−8.46		−8.69	
ΔΔG_bind_	2.04		1.45		2.18	

a All energies are in kcal·mol^−1^,

b ΔEnergy = Energy^complex^–Energy^A-FABP^–Energy^inhibitor^,

ΔG^exp^ were derived from the experimental values in Ref (Barf et al. 2009) using the equation ΔG≈–RTlnIC50,

ΔΔG_bind_ = ΔG^mutant^–ΔG^complex^.

Table 1 lists the individual contributions to the binding free energies. Here, the contributions involve the intermolecular Coulomb and van der Waals interaction energies, ΔE_c_ and ΔE_vdw_, the reaction energy ΔG^R^ and the change in the molecular surface area upon binding (γ·ΔMSA). The contributions favoring binding are those from the van der Waals interactions between binding partners (−33.29 to −41.61 kcal·mol^−1^), the intermolecular Coulomb interactions (−52.04 to −59.51 kcal·mol^−1^) and the contributions from the changes in the molecular surface (−7.55 to −8.79 kcal·mol^−1^). The reaction energies range from 40.84 to 45.88 kcal·mol^−1^, and this term impairs the inhibitor bindings.

The reaction energy related to the desolvation of polar groups always unfavors inhibitor bindings, which is also found in other works [48]–[50]. As seen in Table 1, the unfavorable reaction energy of polar groups is partially compensated by the favorable intermolecular Coulomb interaction. Additionally, intermolecular van der Waals interactions also provide partial compensation to this unfavorable effect.

**Figure 5:**
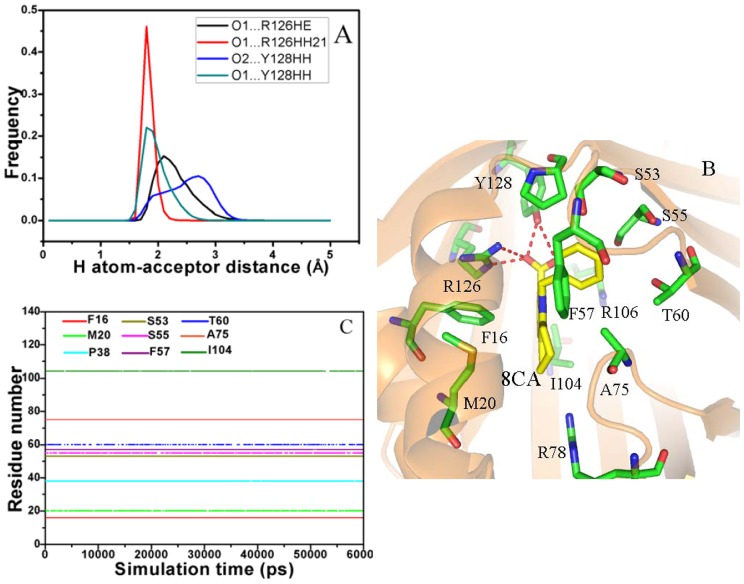
Interactions of key residues in A-FABP with the inhibitor 8CA. Fig. A represents frequency distribution of the H atom…acceptor distance, Fig. B depicts the position of inhibitor 8CA relative to key residues, Fig. C shows the hydrophobic contacts as a function of the simulation time.

In the case of the 8CA/A-FABP complex, the intermolecular Coulomb interaction between 8CA and A-FABP is −57.40 kcal·mol^−1^, which provides an important contribution to the binding. This interaction should include contributions from hydrogen bonds and other polar interactions between 8CA and A-FABP. To clarify this issue, we analyze the hydrogen bonds between 8CA and A-FABP based on the lowest energy structure from MD simulation. The results show that the carboxyl oxygen O1 of 8CA can form three hydrogen bonds with the residues R126 and Y128, while another oxygen atom O2 of the carboxyl also builds a hydrogen bond with Y128 (Fig. 5B). The hydrogen atom-acceptor distance was calculated and their frequency distribution was shown in Fig. 5A. One can see that the distribution of the hydrogen bond O1…R126HH21 has one peak around 1.81 Å and the range of the distribution is narrow. The distribution peaks of the other three hydrogen bonds O1…R126HE, O2…Y128HH and O1…R128HH21 are around 2.11, 2.60 and 1.82 Å, respectively, and the ranges of distribution are wider than the hydrogen bond O1…R126HH21. This result suggests the hydrogen bond O1…R126HH21 is the strongest among these four hydrogen bonds. In order to quantificationally estimate the strength of the hydrogen bonds, the contributions of the hydrogen bonds to the binding free energy of 8CA to A-FABP was calculated using the following equation [58], [59], and the results were listed in Table 2.
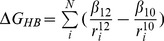
(3)where 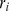 is the H atom-acceptor distance for the *i*th hydrogen bond. The calibrated parameters 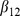 and 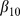 were set to 5.571 and 668.580, respectively [58], [59]. As seen from Table 2, the strengths of the hydrogen bond O1…R126HH21 and O1…R128HH21 are −2.50 and −1.81 kcal·mol^−1^, respectively, while the another two hydrogen bonds are weaker than the two previous hydrogen bonds. These four hydrogen bonds provide a total contribution of −5.76 kcal·mol^−1^ to the binding free energy of 8CA to A-FABP.

**Table 2:** Hydrogen bonding energy calculated based on an empirical equation.

Complex	Hydrogen bonding	HB energy (kcal/mol)
8CA-A-FABP	O2…R126HE	−0.76±0.11
	O1…R126HH21	−2.50±0.19
	O1…Y128HH	−1.81±0.20
	O2…Y128HH	−0.69±0.12
F8A-A-FABP	O12…R126HE	−1.17±0.14
	O11…R126HH21	−1.77±0.16
	O11…Y128HH	−0.46±0.10
	O12…Y128HH	−2.05±0.18
I4A-A-FABP	O26…R126HE	−1.34±0.13
	O27…R126HH21	−1.80±0.13
	O27…Y128HH	−0.51±0.10
	O26…Y128HH	−2.60±0.15
	H18…S55OG	−0.55±(0.12)

The inhibitor 8CA has a negative charge and can interact favorably with the positively charged residues. The previous work from Barf et al. showed that the inhibitors can produce strong polar interaction with A-FABP [17]. To quantificationally evaluate these interactions, the inhibitor-residue polar interaction was computed using the following equation, and the results were listed in Table 3.
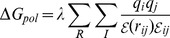
(4)in which 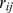 is the internuclear distance between the *i*th atom in the receptor (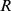) and the *j*th atom in the inhibitor (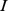). The 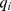 and 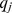 represent the point charges of atoms. The parameter 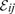 is the distance-dependent dielectric constant determined by using the same function implemented in the program Autodock 3.0.5 [60], and the parameter 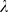 was set to 1.558 in this work [58]. Table 3 shows that 8CA can produce strong polar interactions with the positively charged residues R78, R106 and R126 that correspond −5.93, −10.21 and −21.86 kcal·mol^−1^, respectively, in which R126 is the strongest. The above two analyses suggest that R126 can not only form two hydrogen bonding interactions, but also generate strong charge-charge interaction with the carboxyl of 8CA. This result well explains the cause for the disappearance of the correlated motion of the region R1 relative to the N terminus (Fig. 3) and the reduction in the RMSF values of the residues 122–130 (Fig. 4) after the 8CA binding.

**Table 3:** The polar interactions between inhibitors and the key residues (kcal/mol).

Inhibitors	Residues	Polar interaction (Wild)	Polar interaction (R126A)
8ca	R78	−5.93±0.45	−6.20±0.38
	R106	−10.21±0.47	−14.12±0.24
	R126	−21.96±0.72	−0.10±0.05
F8A	R78	−6.08±0.31	−6.99±0.41
	R106	−8.01±0.52	−13.30±0.47
	R126	−20.01±0.66	−0.01±0.07
I4A	R78	−7.56±0.35	−6.45±0.28
	R106	−10.43±0.42	−14.78±0.31
	R126	−23.26±0.51	−0.05±0.04

According to Table1, besides the intermolecular Coulomb interactions, 8CA also produces favorable van der Waals interaction (−33.35 kcal·mol^−1^) with A-FABP. In order to recognize the contributions of separate residues to van der Waals interaction, the LIGPLOT program was applied to perform the statistical analysis of hydrophobic contacts between 8CA and A-FABP, and a function of the hydrophobic contacts as simulation time was displayed in Fig. 5C [61]. The results show that the residues F16, P38, S53, F57 and A75 form stable hydrophobic contacts with 8CA. Fig. 5C also implies that the hydrophobic contacts of the residues M20, S55, T60 and I104 with 8CA disappear in some conformations. Overall, these nine residues provide main contributions to the van der Waals interaction between 8CA and A-FABP. The above analyses basically agree with the experimental results from Barf et al [17].

**Figure 6:**
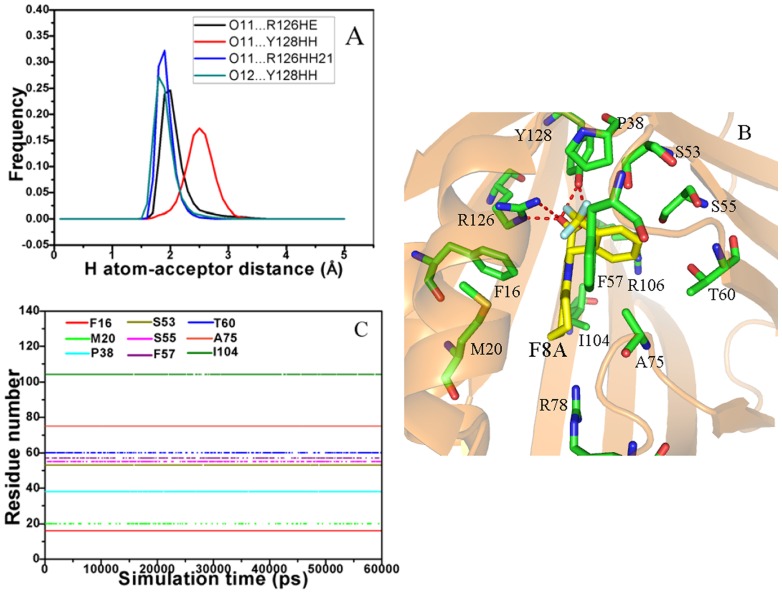
Interactions of key residues in A-FABP with the inhibitor F8A. Fig. A represents frequency distribution of the H atom…acceptor distance, Fig. B depicts the position of inhibitor F8A relative to key residues, Fig. C shows the hydrophobic contacts as a function of the simulation time.

For the F8A/A-FABP complex, the intermolecular Coulomb interactions of F8A with A-FABP is −52.04 kcal·mol^−1^. This interaction mainly comes from the contributions of hydrogen bonds (Fig. 6A) and polar interactions (Table 3). The frequency distribution of the H atom-acceptor distance (Fig. 6A and B) indicates that the carboxyl of F8A forms four hydrogen bond interactions with R126 and Y128, which is similar to the inhibitor 8CA. These four hydrogen bonds include O12…R126HE, O11…R126HH21, O11…Y128HH and O11…Y128HH, and the calculational results based on the equation 3 show that their corresponding strength are −1.17, −1.77, −0.46 and −2.05 kcal·mol-1, respectively. These four hydrogen bonds provide a total contribution of −5.45 kcal·mol^−1^ to the F8A binding. The charge-charge interactions of F8A with the positively charged residues R78, R106 and R126 computed by using the equation 4 were −6.08, −8.10 and −20.01 kcal·mol^−1^, respectively. According to Table 1, the van der Waals interaction between F8A and A-FABP is −33.29 kcal·mol^−1^. The results from the LIGPLOT program (Fig. 6C) reveal that van der Waals interaction mainly rises from the hydrophobic contacts of F8A with the residues F16, M20, P38, S53, S55, F57, T60, A75 and I104. Compared to the 8CA/A-FABP complex, although the van der Waals interactions between F8A and protein hardly change, the intermolecular Coulomb interaction is decreased by −5.36 kcal·mol^−1^. Table 2 suggests that four hydrogen bonds only provide a total contribution of −0.31 kcal·mol^−1^ to the decrease in the intermolecular Coulomb interactions. The information from Table 3 indicates that the total charge-charge interaction between F8A and three charged residues was shifted −4.0 kcal·mol^−1^ relative to 8CA. This issue can be addressed by comparison of the difference in the structures of 8CA and F8A. Structurally, F8A is the derivate of the trifluoromethyl substitution in the position 2 of phenyl group of 8CA. This substitution increases the size of F8A, and correspondingly changes the shape of the binding pocket. Thus these changes induce the alternation of the position and orientation between polar atoms, which in turn decreases the contributions of hydrogen bonds and the intermolecular Coulomb interactions to the binding free energy.

**Figure 7:**
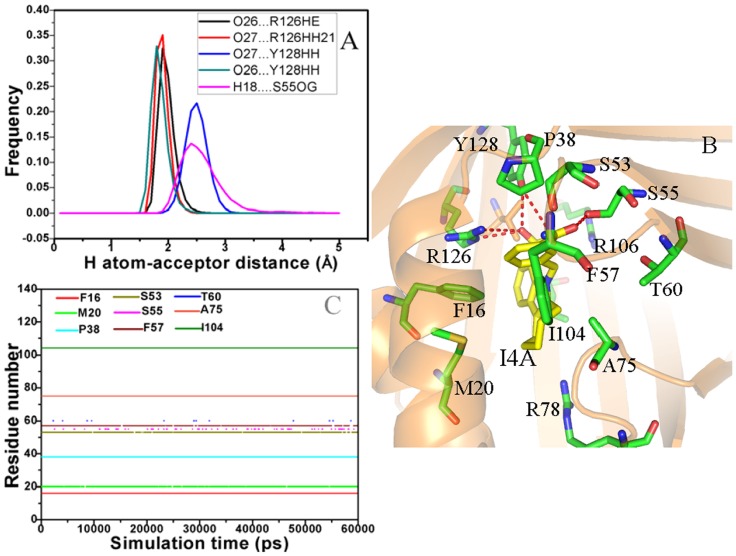
Interactions of key residues in A-FABP with the inhibitor I4A. Fig. A represents frequency distribution of the H atom…acceptor distance, Fig. B depicts the position of inhibitor I4A relative to key residues, Fig. C shows the hydrophobic contacts as a function of the simulation time.

In the case of the I4A/A-FABP complex, the intermolecular Coulomb interaction of I4A with A-FABP is −59.51 kcal·mol^−1^, which is mainly owed to the hydrogen bond interactions of I4A with S55, R126 and Y128 and the charge-charge interactions between I4A and the positively charged residue R78, R106 and R126. The frequency distribution of the H atom-acceptor distance in Fig. 7A reveals that the carboxyl of I4A forms four hydrogen bond interactions with R126 and Y128, which include O26…R126HE and O27…R126HH21 and Y128 O27…Y128HH and O26…Y128HH. Additionally, a weak hydrogen bond (H18…S55OG) also exists between the residue S55 and the hydroxyl of I4A (Fig. 7B). According to Table 2, these five hydrogen bonds give a total contribution to −6.80 kcal·mol^−1^ to the binding free energy. Table 3 shows that the charge-charge interactions of I4A with R78, R106 and R126 also provide a total contribution of −41.25 kcal·mol^−1^ to I4A binding, especially R126 produces the strongest interaction (−23.26 kcal·mol^−1^). As seen from Fig. 7C, I4A can generate stable hydrophobic contacts with the residues F16, M20, F16, P38, S53, F57, A75 and I104, which are mainly responsible for the van der Waals interaction of −41.61 kcal·mol^−1^ between I4A and A-FABP. Compared to the 8CA/A-FABP complex, the intermolecular Coulom and van der Waals interactions are increase by −2.11 and −8.26 kcal·mol^−1^, respectively. The cause resulted in these changes should be the structural difference between I4A and 8CA. According to Fig. 1, the substitution in the position 5 of benzyl group and the seven-membered ring R1 of the scaffold increase the size of I4A. These two structural changes induce the rearrangement of the residues nearby and lead to the changes of interactions, which is supported by the changes of the hydrophobic contacts (Fig. 6C), hydrogen bond energies and charge-charge interactions (Table 2 and 3) and the increase of hydrophobic effect γ·ΔMSA in the molecular surface area upon binding.

Based on the above analyses, two interesting discoveries were obtained: (1) All of three inhibitors form stable hydrophobic contacts with the residues F16, M20, P38, S53, F57, A75 and I104, moreover, their carboxyl produce strong polar interaction with R78, R106, R126 and Y128. These residues just locate at the range corresponding to the obvious decrease in the RMSF (except for the residue 85–95) and the significant alternation of correlated motion. Especially, the polar interaction between inhibitors and R16 and Y128 produces a significant effect on the correlated motion of the C terminus (the range R1) relative to the N terminus, and favors the stability of the C terminus of A-FABP. (2) Both the substitutions of the position 2 and 5 in the benzyl of the common scaffold induce the rearrangement of the residues nearby them. However, the results are different, the substitution of the position 2 reduces the binding affinity, while the substitution of the position 5 and the seven-membered ring of the scaffold strengthen the inhibitor bindings, which basically agrees with the experimental results of Barf et al [17]. Thus, this study provides an important guidance: the inhibitor 8CA can be considered as a valuable starting point of design and optimization for potent and novel inhibitors targeting A-FABP. The seven-membered ring of the scaffold improves affinity, while the substitution in the position 5 of the scaffold also tends to form hydrogen bond interactions with the serine residues S53 and S55. Direct optimizations of the substitution of other polar groups in the position 5 and the seven-membered ring are an efficient means to develop potent inhibitors treating some diseases related to lipid-mediated biology.

### Computational Alanine Scanning

To confirm the significant role of R126, a computational alanine scanning mutagenesis was performed on R126. Table 1 and Table 3 list the changes in the binding free energies of the three inhibitors to A-FABP induced by the mutation R126A. Positive numbers in 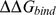 means highly unfavorable mutation, while negative 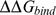 indicates the preference for the alanine residue at the mutated position. The results suggest that R126A leads to the decrease in binding free energy, and produces a significant effect on the intermolecular Coulomb interaction and reaction energy.

According to Table 1, the intermolecular Coulomb interactions of 8CA, F8A and I4A with mutated protein are decreased by 36.56, 30.70 and 31.43 kcal·mol^−1^ relative to the wild complex, respectively, among which the effect of R126A on the 8CA binding is the most significant. However, the results from Table 1 show that R126A produces almost same reduction in the reaction energy, the corresponding reductions for 8CA, F8A and I4A are 18.88, 18.39 and 18.44 kcal·mol^−1^, respectively. Table 3 indicates that the charge-charge interactions between R126A and inhibitors are almost lost relative to the wild complex, which provides the most contribution to the decrease of the intermolecular Coulomb interactions. Additionally, R126A also results in the loss of two hydrogen bonds between the side chain of R126 and the carboxyl of inhibitors, which also gives partial contribution the decrease of the intermolecular Coulomb interactions.

In addition, the mutation R126A induces obvious increase of the intermolecular Coulomb interactions of R106 with inhibitors and the slight reduction of van der Waals interaction between inhibitors and A-FABP. The cause is that R126A decreases the intermolecular Coulomb interactions and the numbers of hydrogen bonds, which in turn results in the loss of intermolecular restrictions and induce the change of the interatomic position. Based on the above analyses, the polar interactions of R126 with the three inhibitors play a key role in the bindings of inhibitors to A-FABP and favors stability of the binding complex.

### Conclusions

In the present work, the binding modes of the three inhibitors 8CA, F8A and I4A to A-FABP were studied by using a combination of 60-ns MD simulation in explicit water and SIE method. Our results show that two substitutions generate different effect on the inhibitor bindings, the substitution in the position 5 of *N*-benzyl and the seven-membered ring of the scaffold strengthen the inhibitor binding, while the substitution in the position 2 of *N*-benzyl make the inhibitor binding weak. The results from computational alanine scanning and polar interaction calculations confirm that the polar interaction of the positively charged residue R126 with the carboxyl of inhibitors plays an important role in the inhibitor bindings. The cross-correlation analysis and RMSF calculation suggest that the polar interactions of the three inhibitors with R126 and Y128 produce significant effects on the internal dynamics of A-FABP, and favor the stabilities of the binding complexes. This study is helpful for the rational design of potent inhibitors within the fields of metabolic disease, inflammation and atherosclerosis.
